# The Occurrence of Malignancy in *Trypanosoma brucei brucei* by Rapid Passage in Mice

**DOI:** 10.3389/fmicb.2021.806626

**Published:** 2022-01-11

**Authors:** Xiao-Li Cai, Su-Jin Li, Peng Zhang, Ziyin Li, Geoff Hide, De-Hua Lai, Zhao-Rong Lun

**Affiliations:** ^1^MOE Key Laboratory of Gene Function and Regulation, State Key Laboratory of Biocontrol, School of Life Sciences, Sun Yat-sen University, Guangzhou, China; ^2^Department of Microbiology and Molecular Genetics, McGovern Medical School, University of Texas Health Science Center at Houston, Houston, TX, United States; ^3^Biomedical Research Centre, School of Science, Engineering and Environment, University of Salford, Salford, United Kingdom

**Keywords:** *Trypanosoma brucei*, dedifferentiation, *in vivo* rapid passage, malignancy, transcriptome

## Abstract

Pleomorphic *Trypanosoma brucei* are best known for their tightly controlled cell growth and developmental program, which ensures their transmissibility and host fitness between the mammalian host and insect vector. However, after long-term adaptation in the laboratory or by natural evolution, monomorphic parasites can be derived. The origin of these monomorphic forms is currently unclear. Here, we produced a series of monomorphic trypanosome stocks by artificially syringe-passage in mice, creating snapshots of the transition from pleomorphism to monomorphism. We then compared these artificial monomorphic trypanosomes, alongside several naturally monomorphic *T. evansi* and *T. equiperdum* strains, with the pleomorphic *T. brucei*. In addition to failing to generate stumpy forms in animal bloodstream, we found that monomorphic trypanosomes from laboratory and nature exhibited distinct differentiation patterns, which are reflected by their distinct differentiation potential and transcriptional changes. Lab-adapted monomorphic trypanosomes could still be induced to differentiate, and showed only minor transcriptional differences to that of the pleomorphic slender forms but some accumulated differences were observed as the passages progress. All naturally monomorphic strains completely fail to differentiate, corresponding to their impaired differentiation regulation. We propose that the natural phenomenon of trypanosomal monomorphism is actually a malignant manifestation of protozoal cells. From a disease epidemiological and evolutionary perspective, our results provide evidence for a new way of thinking about the origin of these naturally monomorphic strains, the malignant evolution of trypanosomes may raise some concerns. Additionally, these monomorphic trypanosomes may reflect the quantitative and qualitative changes in the malignant evolution of *T. brucei*, suggesting that single-celled protozoa may also provide the most primitive model of cellular malignancy, which could be a primitive and inherent biological phenomenon of eukaryotic organisms from protozoans to mammals.

## Introduction

*Trypanosoma brucei* has a complicated life cycle, with multiple differentiation and multiplication events in both the mammalian host and the insect vector (pleomorphism), which limits its distribution in Africa to where the tsetse fly vector is present (Wijers, 1959; Vickerman, 1965). *T. brucei* belongs to the subgenus *Trypanozoon* in which *T. evansi* and *T. equiperdum* are also included (Brun et al., 1998). Unlike *T. brucei*, *T. evansi* and *T. equiperdum* cannot undergo the complete life cycle through the tsetse fly due to their inability to transform into the procyclic form (one of the key stages in the vector) and have adapted, respectively, to mechanical and venereal transmission. They simply retain the proliferative slender forms (monomorphism) which is equivalent to the *T. brucei* long slender (LS) form, in the mammalian bloodstream. *T. evansi* and *T. equiperdum*, the pathogens of surra in livestock and dourine in horses, have long been considered as mutants of *T. brucei* (Claes et al., 2005; Li et al., 2006; Lai et al., 2008; Desquesnes et al., 2013; Wen et al., 2016). They are globally distributed, including in Africa, Asia, South America and some regions of Europe, and have radiated far more successfully than *T. brucei* (Lun et al., 2010; Schnaufer, 2010). Interestingly, parallels can be drawn between malignant metazoan cells and these protozoan parasites as they have lost the ability for differentiation and the control of cell numbers. Based on a large number of biological and genomic characteristics of these parasitic protozoa and a comparison with characteristics of malignant tumor cells (Sachs, 1986; Tenen, 2003; Murgia et al., 2006; Rebbeck et al., 2009; Hanahan and Weinberg, 2011; Carnes et al., 2015), it has been suggested that *T. evansi* and *T. equiperdum* originated as the result of malignancy in *T. brucei* cells that are now found in nature (Lun et al., 2015). However, it has not been systematically established whether this phenomenon of malignancy is widespread in single-celled protozoa, represented by trypanosomes, and, if so, how such malignancy is triggered.

Pleomorphism in the life cycle stages of *T. brucei* is crucial to its transmission by the tsetse fly, and multiple cell differentiation and development events must be involved. Thus, the distinct *T. brucei* life cycle forms are specialized for specific host niches and have evolved to optimize parasite survival and transmission. In the mammalian host, one of the bloodstream forms (BSF) of *T. brucei*, the long-slender (LS) form (herein referred to as BSF-LS) undergoes a differential transition at a critical parasite density, resulting in a shortening of the cell body and flagellum to form a short stumpy (SS) form (herein referred to as BSF-SS) (Black et al., 1985; Vassella et al., 1997). These morphological transitions are also accompanied by a range of cellular events such as cell cycle regulation, biochemical and physiological changes, reorganization of organelle and cell structure, changes in stage-specific surface protein expression and synthesis of mitochondrial respiratory enzymes for pre-adaptation to aerobic metabolism and energy utilization (Vickerman, 1965; Dean et al., 2009; Rico et al., 2013; Silvester et al., 2017). These changes enable the two forms to perform different functions at each life cycle stage, as well as allowing discrimination between them. Specifically, the population of BSF-LS form is committed to establishing an infection through rapid proliferation in the host while the BSF-SS form has evolved to not only extend longevity of the host but to maximize the potential for parasite cyclical transmission (MacGregor et al., 2012).

Generally, pleomorphic trypanosomes, which generate both slender and stumpy forms, are found in nature (Wijers, 1959; Vickerman, 1965). However, extensive passage of *T. brucei* BSF in either mice or culture, under laboratory conditions, results in the selection of rapidly growing monomorphic parasites. These remain as dividing BSF-LS forms until the death of their host (Ashcroft, 1960; Turner, 1990; McWilliam et al., 2019). These laboratory-generated monomorphic trypanosomes are incapable of arresting as the BSF-SS form. In nature, *T. evansi* and *T. equiperdum* both behave as monomorphic trypanosomes suggesting parallels between laboratory selected trypanosomes and these naturally evolved pathogens. The mechanism of generating monomorphism in the laboratory or nature is poorly understood. One possibility is that the accumulation of stochastic mutations interferes with the differentiation process and contributes to the generation of monomorphism.

A variety of important studies have provided insights into the understanding of the mechanisms that drive *T. brucei* cellular differentiation and transmission. Notably, the density-dependent stumpy induction factor (SIF) signal reception and transduction via the cAMP pathway, which promotes the differentiation of BSF-LS to SS forms, has been most systematically studied (Mancini and Patton, 1981; Vassella et al., 1997; Mony et al., 2014). This has also been termed the quorum sensing (QS) signaling pathway as it appears to regulate parasite density in the host. A systematic dissection of the downstream factors in the QS regulation pathway has identified over 30 molecules involved in detecting the differentiation stimulus, signal propagation and the implementation of cellular changes (Mony et al., 2014). Some of the factors have been verified as requirements for stumpy formation in a series of subsequent studies. These include the serine/threonine phosphatase (PP1), the RNA binding protein (RBP7), AMP-activated kinase (AMPK), Dyrk/YAK kinase (YAK), a member of the NEK kinase family (NEK17) and have been placed in a non-linear hierarchy in the QS pathway (Saldivia et al., 2016; McDonald et al., 2018). Additionally, other pathways that are independent of QS mediation have also been explored, such as the transcriptional attenuation of the variant surface glycoprotein (VSG) expression site (ES) linked to antigenic variation (Zimmermann et al., 2017), as well as some negative regulators, for example the repressor of differentiation kinases (RDK1/2) (Jones et al., 2014), the regulator of ESAG9 factor 1 (REG9.1) (Rico et al., 2017), and others (Barquilla et al., 2012; Szöőr et al., 2020). Furthermore, “inducible monomorphic” trypanosomes can also be produced from pleomorphic ones by genetic modification of the differentiation regulators mentioned above. However, as far as we know, this has not been reported to occur spontaneously.

Despite a great deal of work that has uncovered many key nodes in the differentiation regulation pathway, direct links have not been established between these regulators and the spontaneous formation of monomorphism in trypanosomes either in the laboratory or in nature. Furthermore, the evolution of monomorphism in protozoan trypanosomes may represent a primitive type of cell malignancy, the evolution of this process, in nature, may have enabled the successful radiation of forms like *T. evansi* and *T. equiperdum*, which has not yet been realized. In this study, by continuous passage of *T. brucei* in mice, we have successfully established a series of populations of trypanosomes ranging from pleomorphic to monomorphic, in the laboratory. We have conducted, for the first time, a comparative transcriptomic analysis between a pleomorphic *T. brucei* and a series of monomorphic trypanosome stocks obtained from both laboratory simulation (*T. brucei*) and natural evolution (*T. evansi* and *T. equiperdum*). Detailed analysis has thrown new light on the generation of monomorphism in trypanosome cells and shows that it is a cumulative process of quantitative change leading to qualitative change and, in an analogous manner to the occurrence of malignancy in metazoan organisms, both lose their directional differentiation and proliferate indefinitely.

## Materials and Methods

### Animals

Healthy female Swiss mice (aged 6∼8 weeks) and SD rats (aged ∼8 weeks) were purchased from the Laboratory Animal Center of Sun Yat-sen University. Animal care and experimental procedures complied with the Chinese Laboratory Animal Administration Act (2017). All animal experiments were conducted in accordance with protocols approved by the Laboratory Animal Use and Care Committee of Sun Yat-sen University under the license no 31720103918. Animal studies were reported according to the ARRIVE guidelines.

### Trypanosomes

The strains of *Trypanosoma brucei*, *T. evansi*, and *T. equiperdum*, used in this study are listed in Supplementary Figure S1. The pleomorphic *T. brucei* AnTat1.1 (EATRO1125) strain (Delauw et al., 1985) was used for establishing monomorphic cell lines in this study. Parasites were thawed from liquid nitrogen and inoculated intraperitoneally into mice. Specifically, parasites were cloned *in vitro* to establish a genetically clonal population prior to continued passaging *in vivo*. Parasitaemia was measured, from tail blood using a hemocytometer, starting from day 2 post-infection and subsequently daily. When the trypanosome density had increased to about 10^7^∼10^8^ cells ml^–1^, it was used for further sub-inoculation into two naive mice, each received a total of 200 μl of phosphate buffered saline (PBS) with 10^5^ trypanosomes. Repeatedly, we performed a total of 120 successive passages in mice at 2- to 3-day intervals. Trypanosome stocks were isolated at intervals during these passages and named appropriately (e.g., Tbp40 refers to a stock obtained from passage 40). For rat infections, 10^3^ parasites were inoculated by subcutaneous injection to establish chronic infections.

Trypanosomes were isolated from the blood of infected mice by DEAE cellulose (DE-52) as described (Lanham and Godfrey, 1970). After the last centrifugation, the trypanosome sediment was frozen at -80°C for RNA-seq preparation. Using the same method, parasites isolated under sterile conditions were used for *in vitro* culture.

The highly concentrated trypanosome suspension in PSG was diluted to 2 × 10^5^ cells ml^–1^ in Hirumi’s modified Iscove’s medium 9 (HMI-9), complemented with 10% (v/v) heat-inactivated fetal bovine serum (FBS; Gibco) and incubated at 37°C and 5% CO_2_ for *in vitro* culture (Hirumi and Hirumi, 1989).

### Morphology and Cell Cycle Analysis

The blood of infected mice (*in vivo*) was smeared onto slides, left to dry and fixed in methanol. Methanol-fixed thin blood smears were stained with Giemsa. Then, the Giemsa-stained thin blood smears were analyzed on a Nikon Eclipse Ni-U microscope to record cell morphology, kinetoplast (K) and nucleus (N) configuration counts.

### *In vitro* Differentiation to Procyclic Forms

Procyclic differentiation assays refer to the previous literature with modifications as described (McDonald et al., 2018). Pleomorphic *T. brucei* (Tbp1) parasites, laboratory-adapted monomorphic passages (includingTbp40, Tbp80, Tbp120) and monomorphic *T. evansi* and *T. equiperdum* parasites, were purified from whole blood and resuspended at 2 × 10^6^ cell ml^–1^ in SDM-79 + 10% FBS (v/v) media supplemented with 6 mM cis-aconitate (Sigma) to induce procyclic differentiation. These were then incubated at 27°C in 5% CO_2_. After 1, 6, 12, 24, or 48 h cultivation (depending on the strains), 1 ml of the cell culture was concentrated at 1000 g, and then air-dried smears were prepared from the parasite pellets and fixed in 1% paraformaldehyde for stage-specific procyclin coat expression analysis by immunofluorescence. Meanwhile, CA was removed from the medium after 6- or 24-h treatments and the parasites were used for subculture *in vitro*.

### Immunofluorescence Analysis

To determine the efficiency of synchronous differentiation of trypanosomes from BSF to PCF *in vitro*, differentiation was assessed by following the expression of the procyclic stage-specific procyclin coat using immunofluorescence microscopy, as described previously (McDonald et al., 2018). Parasite smears were fixed in pre-cooled methanol, rehydrated in 1 × PBS for at least 5 min. Excess liquid was removed, slides were then incubated for 1 h at room temperature with the anti-procyclin primary antibody (Cedarlane, Canada) diluted at 1:1000 in PBS. Then smears were washed 5 times in 1 × PBS, each time for 5 min and incubated for 30 min at 37°C with the anti-mouse secondary antibody coupled to an Alexa 594 fluorophore (Invitrogen). The secondary antibody was removed and the smears were washed as before. Smears were then finally stained with DAPI for visualization of the kinetoplast and nuclear DNA contents and observed with a DM4 B epifluorescence microscope (Leica) and the images captured with a DFC900 GTC camera (Leica).

### *In vitro* 8pCPT-cAMP Resistance Assays

Assays were performed as described previously (Mony et al., 2014). Parasites isolated from mice were seeded at 10^5^ cells ml^–1^ in the flasks. After 2∼3 passages of adaptation *in vitro*, each flask was split, one being exposed to 100 mM of 8pCPT-cAMP (Abcam) while the others were left uninduced. Assays were performed in triplicate, growth being monitored every 24 h. Cells were maintained at ≤2 × 10^6^ cells ml^–1^ using HMI-9, supplemented with 8pCPT-cAMP where needed.

### RNA Isolation and Quantitative Real-Time PCR

For RNA preparation, parasites were lysed in 1 ml TRIzol (Invitrogen), with a maximum of 5 × 10^8^ cells ml^–1^ TRIzol. Total RNA was isolated as described previously (Liao et al., 2014). The purity and concentration of the resultant RNA was measured on a Nanodrop spectrophotometer, then stored at -80°C.

After removal of genomic DNA by adding gDNA Eraser, 1 μg of total RNA was used as a template to generate cDNA in a reverse transcriptase reaction with random hexamers using a PrimeScript™ RT reagent Kit (Takara, Japan). Following cDNA synthesis, qPCR reactions were set up using the LightCycler^®^ 480 SYBR Green I Master mix (Roche, Switzerland) by following the manufacturer’s instructions. The specificity and dimer contamination of each primer pair were confirmed (Supplementary Table S1). The control primers used targeted *T. brucei* gene AN1 (Tb927.10.12970) due to its stability during trypanosome life cycle stage (Kabani et al., 2009; Jones et al., 2014). The relative abundance of RNAs was determined by the 2^–ΔΔCt^ method (Pfaffl, 2001).

### Bioinformatics

RNA-Seq was carried out by the Annoroad Gene Technology Corporation (Beijing). The Illumina cDNA libraries were prepared using the TruSeq library preparation protocol with poly(A)-selection. Sequences were done on an Illumina NovaSeq 6000 platform with 150 bp paired-end reads. In order to guarantee the data quality, raw data was trimmed to remove the generic primer sequence. In addition, adapters, contaminated reads, low quality reads (>15% bases with Quality value ≤ 19) and reads with > 5% uninscribed bases (Ns) were also removed. Then the clean reads were aligned to the *T. b. brucei* TREU927 reference genome from the TriTrypDB database^1^. Bowtie2 v2.2.3 was used for building the genome index and clean reads were then aligned to the reference genome using HISAT2v2.1.0 with default parameters - dta - t - p 4. Read counts for each gene in each sample were determined with HTSeq v0.6.0, and FPKM (fragments per kilobase million mapped reads) were then calculated to estimate the expression level of genes in each sample. DEGseq2 was used for differential gene expression analysis between pairwise samples with biological replicates. It was used to estimate the expression level of each gene in each sample using linear regression and then the *p*-value was calculated with the Wald test. Finally, the *p*-Value was corrected by the BH method (Hochberg and Benjamini, 1990). Genes with *q* ≤ 0.05 and fold change (FC) ≥ 1 were identified as differentially expressed genes (DEGs).

The differential expression profiles between the *T. brucei* BSF-LS and BSF-SS, as well as regulators of differentiation processes sourced from published data (Barquilla et al., 2012; Mony et al., 2014; Silvester et al., 2018), were used as validated reference databases for the analysis of the status and differentiation regulation ability of various monomorphic trypanosomes.

### Statistical Analysis

All graphs were produced using GraphPad Prism 8 (GraphPad Software, Inc., San Diego, CA, United States^2^), and R version 4.0.4. Microscope images were normalized and adjusted (including cropping, brightness, contrast, overlay, scale bar application) using Adobe Photoshop 2017.0.0 (Adobe Systems Software, San Jose, CA, United States).

### Data Deposition

All the original transcriptome raw data and clean data from this study were deposited in the GEO repository (GSE184265) https://www.ncbi.nlm.nih.gov/geo/query/acc.cgi?acc=GSE184265.

## Results

### Generation and Verification of Monomorphic Trypanosomes in the Laboratory

**FIGURE 1:**
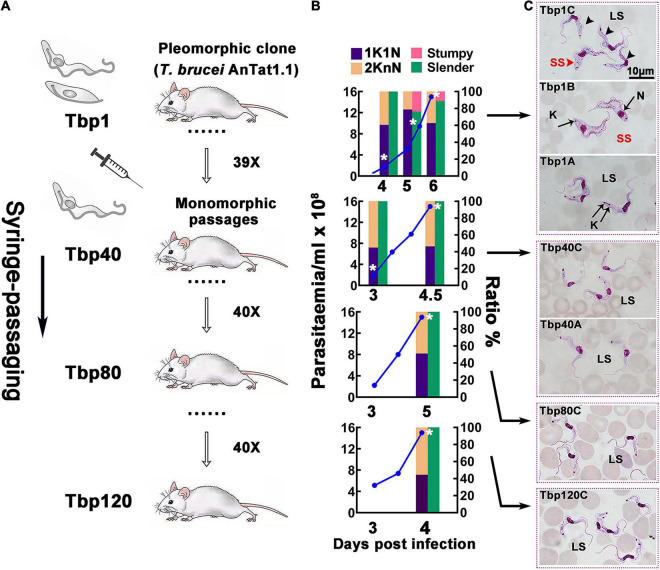
Schematic diagram of the passaging process *in vivo* and the biological characteristics of passaged *T. brucei* cells. **(A)** Schematic diagram of the *in vivo* passaging adopted in this investigation. The pleomorphic *T. brucei* AnTat1.1 strain (Tbp1) was cloned and used as the parent strain for rapid syringe-passaging in mice; the resulting 40th, 80th and 120th generations (Tbp40, Tbp80, and Tbp120) were selected for detailed analysis and further study. **(B,C)** Biological characteristics of different numbers of passages of *T. brucei* in mice. **(B)** Parasitaemia (blue lines) of each passage was monitored after day 2 post-infection; the proportion of morphological slender forms (green bars), stumpy forms (red bars) and parasites presenting 1 kinetoplast & 1 nucleus (1K1N, purple bars), or 2 kinetoplasts & 1 or 2 nuclei (2KnN, orange bars) were assessed in 300 cells at each time point. The cell cycle status with statistics of KN provides a measure of proliferating cells in the population. As the parasitaemia increased beyond ∼4 × 10^8^ cell ml^– 1^, the proportion of parasites with 1K1N in parental Tbp1 ascended, and declined later before the host dies, an indication of the existence of cell cycle-arrested stumpy forms, which was not observed in the Tbp40, Tbp80, and Tbp120 passages. White asterisks indicate when the sample was harvested for RNA-seq. **(C)** Giemsa-stained morphology of passaged *T. brucei* isolated from the parasitemia ranging from 10^8^∼10^9^cells ml^– 1^ in murine infection (depending on the cell line). The source pleomorphic stock, Tbp1, generated morphological stumpy forms (top three panels are sequentially shown from bottom to top as the parasitaemia develops), while Tbp40, Tbp80, and Tbp120 passages generated only slender forms corresponding to the following lower four panels throughout the whole infection, which was consistent with the configurations in panel **(B)**. LS, long slender; SS, short stumpy; N, nucleus; K, kinetoplast.

The pleomorphic *T. brucei* AnTat1.1, which was cloned and confirmed for its differentiation ability *in vivo*, was named Tbp1 and was used for the rapid passaging in mice. As a result, a series of monomorphic passages were produced, collected at the 40th, 80th and 120th generations (here they have been termed Tbp40, Tbp80 and Tbp120; also see Figure 1). The survival of mice infected with Tbp40, Tbp80 and Tbp120 was shortened from 6 days to 4∼5 days compared with the Tbp1 infection (Figure 1B). It was seen that a density dependent transition from morphological bloodstream form-long slender (BSF-LS) to bloodstream form-short stumpy (BSF-SS) and cell cycle arrest in the G0/G1 phase, with an increased proportion of cells with one kinetoplast and one nucleus (1K1N), occurred in Tbp1 infected mice at a parasitemia of 4∼7 × 10^8^ cells ml^–1^. In contrast, such morphological transformations and cell cycle arrest were absent in Tbp40, Tbp80 and Tbp120. Interestingly, Tbp40 and Tbp80, but not Tbp120, could still generate BSF-SS when they were inoculated into rats (Supplementary Figure S2A). The failure of Tbp120 to form stumpy cells was also indirectly demonstrated by its stronger lethality to rats (Supplementary Figure S2B).

**FIGURE 2:**
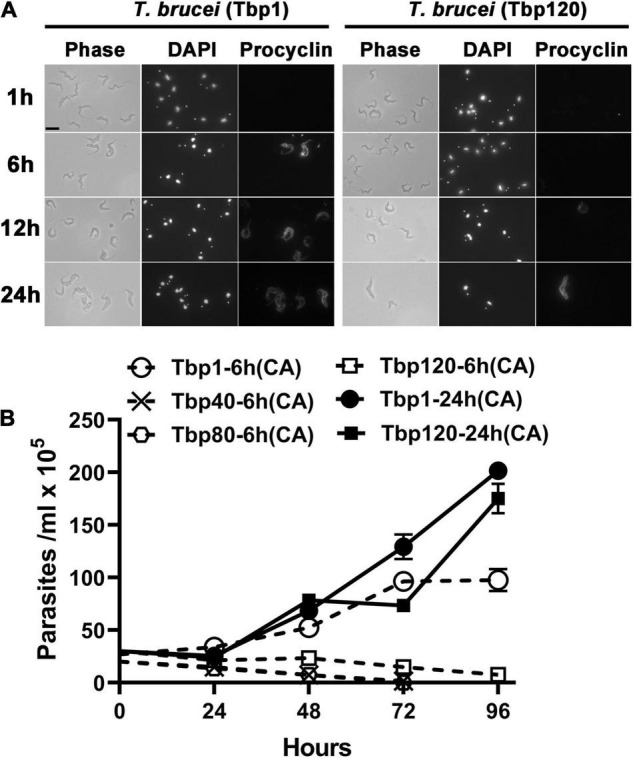
Transformation efficiency of different passage populations of *T. brucei* from bloodstream forms into procyclic forms *in vitro*. **(A)** Expression of the procyclic stage-specific coat procyclin of pleomorphic *T. brucei* (Tbp1) and the Tbp120 trypanosomes treated with cis-aconitate (CA) for different times *in vitro*. Parasites harvested from infected mice at the peak parasitaemia were incubated in SDM-79 medium and exposed to 6 mM CA for 1, 6, 12, and 24 h respectively. The efficiency of transformation was analyzed by measuring the expression of procyclin in procyclic forms (PCF). The Tbp120 trypanosomes (right panel) showed reduced differentiation efficiency than the trypanosomes of Tbp1 (left panel) indicating the absence of stumpy cells at the peak parasitaemia in Tbp120. **(B)** The growth status of trypanosomes after withdrawal of CA from the medium for 6 or 24 h. The Tbp40, Tbp80 and Tbp120 trypanosomes failed to continue to grow in pure SDM-79 after 6 h induction, showing an inefficient transition to PCFs from the entirely slender form population; while the stumpy population existing in Tbp1 trypanosomes could significantly improve the efficiency of transformation into PCFs under the same incubation conditions and could also be cultured *in vitro* for continuous growth. The slender forms in Tbp120 could finally be forced to differentiate into PCF if they were treated with CA for 24 h.

**FIGURE 3:**
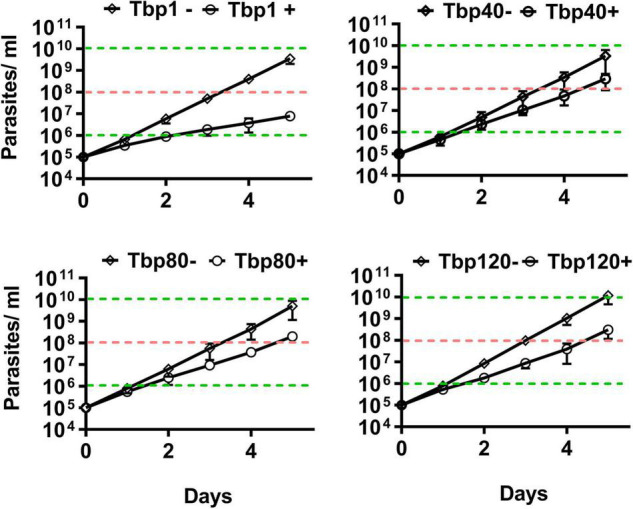
*In vitro* cumulative growth curves of different passaged populations of *T. brucei* in response to the effect of 8pCPT-cAMP. The Tbp40, Tbp80, and Tbp120 trypanosomes isolated from the infected mice showed resistance to 8pCPT- cAMP-mediated growth arrest *in vitro* compared with their parent (Tbp1). After 4 days of induction, the cumulative parasites of Tbp1 grew to about 10^6^ cells ml^– 1^ (indicated by the lower green dotted line), while the Tbp40, Tbp80, and Tbp120 trypanosomes could reach to 10^8^ cells ml^– 1^ (indicated by the pink dotted line); but had similar growth trends in the absence of 8pCPT-cAMP shown for the controls. –, control group; +, 8pCPT-cAMP treated.

Using *in vitro* experiments, it was also indirectly shown that there were only homogeneous BSF-LS at the peak of infection with Tbp40, Tbp80 and Tbp120, due to the reduced efficiency of synchronous differentiation to procyclic forms (PCF) (Figure 2). BSF-SS is known to be more pre-adapted to the tsetse fly than BSF-LS. Immunofluorescence showed that the procyclin protein specifically expressed by PCF was detected in Tbp1 after incubation with cis-aconitate (CA) for just 6 h (Figure 2A), and these treated parasites could grow in CA-free SDM-79 like normal PCFs (Figure 2B). However, such expression was hardly observed in Tbp40, Tbp80 and Tbp120, unless induced for a much longer time (24 h). Furthermore, these monomorphic passages also showed resistance to 8pCPT-cAMP-mediated growth arrest in comparison with Tbp1 (Figure 3). Together, these data confirmed that we have successfully simulated the evolutionary-like process of moving from pleomorphic to monomorphic forms in trypanosomes and obtained a series of phased monomorphic generations derived from pleomorphic *T. brucei*.

### Global View of the Transcriptome in Trypanosomes

To explore the possible developmental mechanisms involved in evolution of pleomorphic to monomorphic forms of trypanosomes, we analyzed the RNA expression profiles of the pleomorphic *T. brucei* and various naturally occurring monomorphic trypanosomes. This included the series of laboratory-adapted monomorphic *T. brucei* populations derived from mouse infection, three strains of *T. evansi* (STIB805, STIB816, and CPOgz) and three strains of *T. equiperdum* (STIB818, STIB841, and STIB842). The latter two species are generally considered to be typically monomorphic trypanosomes that have evolved from *T. brucei* in the field. They have completely lost the ability to adapt to the insect stage and no transformation from the BSF to PCF was observed in the *T. evansi* and *T. equiperdum* strains *in vitro*, even in the SDM-79 medium induced by cis-aconitate, and all perished after 2∼3 days (Supplementary Figure S3). Blood was taken for RNA preparation from each passage at a parasitemia ranging from 10^8^ to 10^9^ cells ml^–1^ as indicated in Figure 1B and parasite morphology was also characterized accordingly (Figures 1B,C). A total of 38 transcriptomes were finally accessed, with all samples having three biological replicates, except for sample Tbp1B (two replicates). Replicates of each sample were correlated within *r* = 0.93∼1 demonstrating minimal experimental variation (Supplementary Figure S4). The percentage of clean reads aligning to the *T. brucei* TREU927 genome varied between 72% and 91% (Supplementary Table S2, Sheet 1). Among them, the overall match rates of *T. evansi* and *T. equiperdum* are better than that of *T. brucei* AnTat1.1.

**FIGURE 4:**
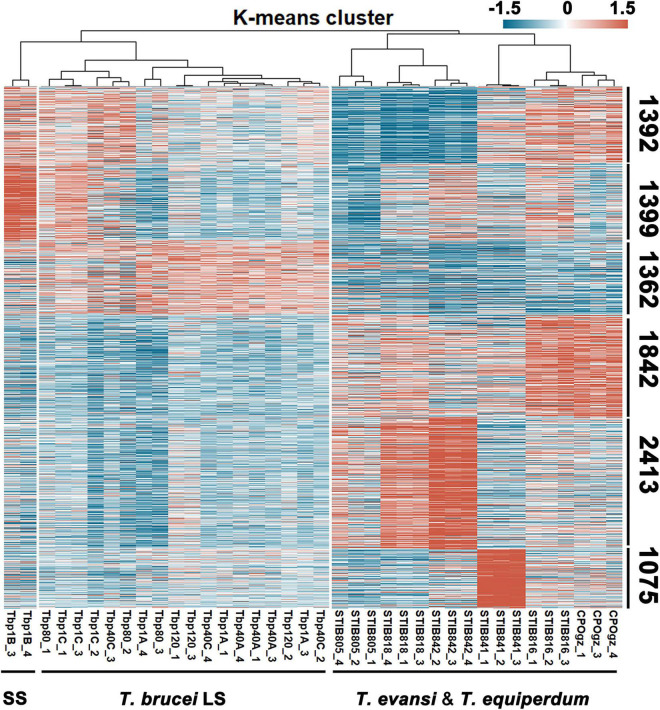
Landscape of the transcriptome of trypanosomes. A total of 9483 genes from 38 trypanosome samples were subjected to K-means analysis. According to the expression patterns in each sample, genes were clustered into 6 clusters as numbered. SS represents *T. brucei* short stumpy form; LS represents the *T. brucei* slender form, including both differentiation-committed and monomorphic slender forms. Detailed notation of sources of *T. brucei* RNA can be found in Figures 1B,C. Detailed RNA abundances for each gene, as estimated by fragments per kilobase million mapped reads (FPKM) in Supplementary Table S2, Sheet 2.

On the basis of hierarchical clustering analysis (Figure 4), the samples (Figures 1B,C for *T. brucei* sample notations) were categorized into three major groups: two branches in *T. brucei*, represent the typical differentiated Tbp1B (BSF-SS) and the rest (all BSF-LS and Tbp1C); the third group comprises *T. evansi* and *T. equiperdum*. Genes were divided into six clusters, based on K-means analysis of transcriptional profiles (Figure 4 and Supplementary Table S2, Sheet 2). Only the genes in the top three clusters displayed distinct patterns between BSF-SS and BSF-LS, suggesting a transcriptional remodeling during the differentiation process, while the bottom three clusters had a constant transcriptional level among them. However, transcriptional patterns in *T. evansi* and *T. equiperdum* were seen to be diverse. The transcripts in cluster 1 are significantly down-regulated in STIB805, STIB818 and STIB842. The cluster 2 genes favored by the BSF-SS were most down-regulated in STIB805. Genes in cluster 3 were down-regulated in all *T. evansi* and *T. equiperdum*. Genes in cluster 4 were exclusively elevated in *T. evansi* strains STIB816 and CPOgz. While, clusters 5 and 6 were represented by different *T. equiperdum* strains, genes in cluster 5 were biased toward STIB818 and STIB842, genes in cluster 6 were biased toward STIB841. Supplementary Figure S5 shows the number of altered genes that are statistically significant (≥2-fold, *q* ≤ 0.05) in pairwise comparisons.

### Assessment of the Pleomorphism of the Parental Passaged Cell Line *Trypanosoma brucei* by Transcriptomic Analysis

The analysis of the two stages in the life cycle showed major changes in transcriptome composition during the transition from the proliferative BSF-LS (Tbp1A) to the BSF-SS cells (Tbp1B and Tbp1C) (Supplementary Figure S5). A total of 364 transcripts were increased in abundance in the Tbp1B, including stumpy-specific markers (such as PAD1, ESAG9s, and PIP39), some pre-transcribed genes of the PCF-stage, for example, PAD2, procyclins, mitochondrial genes (e.g., NADH dehydrogenase subunit 1, cytochrome oxidase subunits). There were 649 genes increased in Tbp1A, including the typical slender-associated genes involved in glycolytic metabolism (such as glyceraldehyde 3-phosphate dehydrogenase, glycerol kinase), cytoskeleton organization (e.g., beta tubulin, proteins associated with flagellar assembly and transport), and cell cycle-regulating genes (e.g., histones and cytokinesis initiation factors) (Supplementary Table S3, Sheet 1). When comparing Tbp1C to either Tbp1A or Tbp1B, only 180 and 145 DEGs were identified, confirming an intermediate proportionate composition of BSF-LS and BSF-SS in sample Tbp1C (Supplementary Figure S5 and Supplementary Table S3, Sheet 1). Therefore, we merged Tbp1B and Tbp1C as the BSF-SS group and revealed a total of 370 up-regulated transcripts and 656 down-regulated in comparison with BSF-LS (Supplementary Table S3, Sheet 2).

**FIGURE 5:**
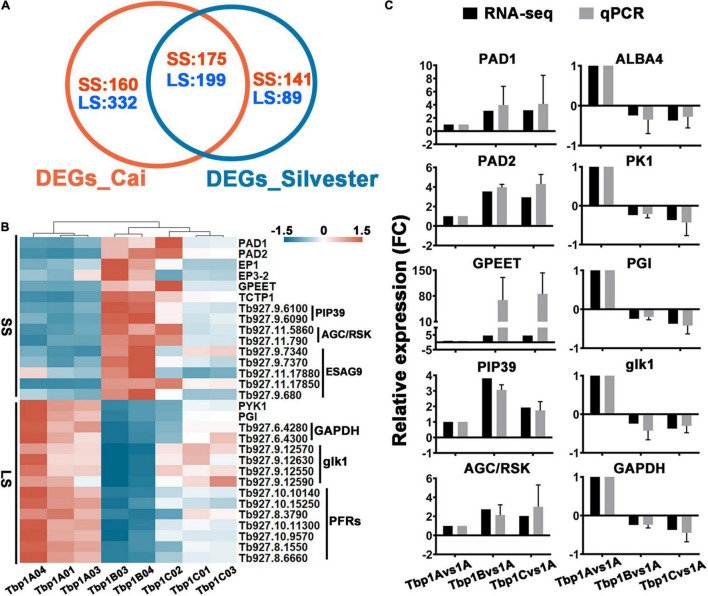
Comparisons of the differential expression between long slender (LS) and short stumpy (SS) forms in two independent studies and assessment of the transcriptomic survey by well characterized differentiation markers. **(A)** Venn diagram displaying differentially expressed gene (≥2-fold, *q* ≤ 0.05) numbers which shows consistent expression trends between *T. brucei* LS and SS as detected by Silvester et al. (2018) and our study. A total of 374 differentially expressed genes with consistent trends were shared in both studies. Although the individual 492 and 230 transcripts in each dataset did not achieve the fold change threshold, they showed concordant expression trends. The blue and red fonts indicate the number of up-regulated genes in the LS and SS, respectively. **(B)** The heat map displays the expression profiles of selected genes in panel **(A)** known to be differentially expressed in the *T. brucei* LS (left panel) and SS (right panel). **(C)** Expression level validation of markers listed in panel **(B)** by qPCR. Relative expression ratio of each gene presenting the fold change value of Tbp1 vs. Tbp1B and Tbp1C, is consistent with the expression trend detected in RNA-seq. The values are mean ± SE (*n* = 3).

We also compared our results to a recently published study from Silvester and colleagues (Silvester et al., 2018 and Supplementary Table S3, Sheet 3), which applied a similar comparison to two stages (BSF-LS and SS) of pleomorphic *T. brucei* grown *in vivo*. A total of 374 transcripts with a FC ≥ 2-fold, *q* ≤ 0.05 were shared in both studies, including 175 up-regulated in BSF-SS forms, 199 down-regulated transcripts (Figure 5A). Although 230 and 492 significantly regulated transcripts were exclusively found in the Silvester et al. (2018) and our study, they still have consistent expression bias trends. These alterations between BSF-LS and BSF-SS overlap well in the two independent studies, both have fully identified specific expression in the life cycle stages of *T. brucei* (Figure 5B). Furthermore, the expression levels of faithful stage specific markers were verified by qPCR (Figure 5C). Finally, we concluded with a total of 1096 stable differentiation characteristic transcripts (DCTs) from the two studies as a solid dataset to distinguish the differentiation state for subsequent analysis (Supplementary Table S3, Sheet 4).

### Expression Differences in Differentiation Characteristic Transcripts Between Differentiation-Committed *Trypanosoma brucei* Slender Forms and Monomorphic Trypanosomes

To gain a new insight into the defective differentiation process, we analyzed the expression patterns of DCTs in all monomorphic trypanosomes, including 4 samples of laboratory-adapted *T. brucei* (Tbp40A, Tbp40C, Tbp80C, and Tbp120C) and 6 naturally monomorphic strains of *T. evansi* and *T. equiperdum*. In general, most monomorphic trypanosomes display a Tbp1A-like pattern of these DCTs, as distinct from the Tbp1B representative of BSF-SS.

**FIGURE 6:**
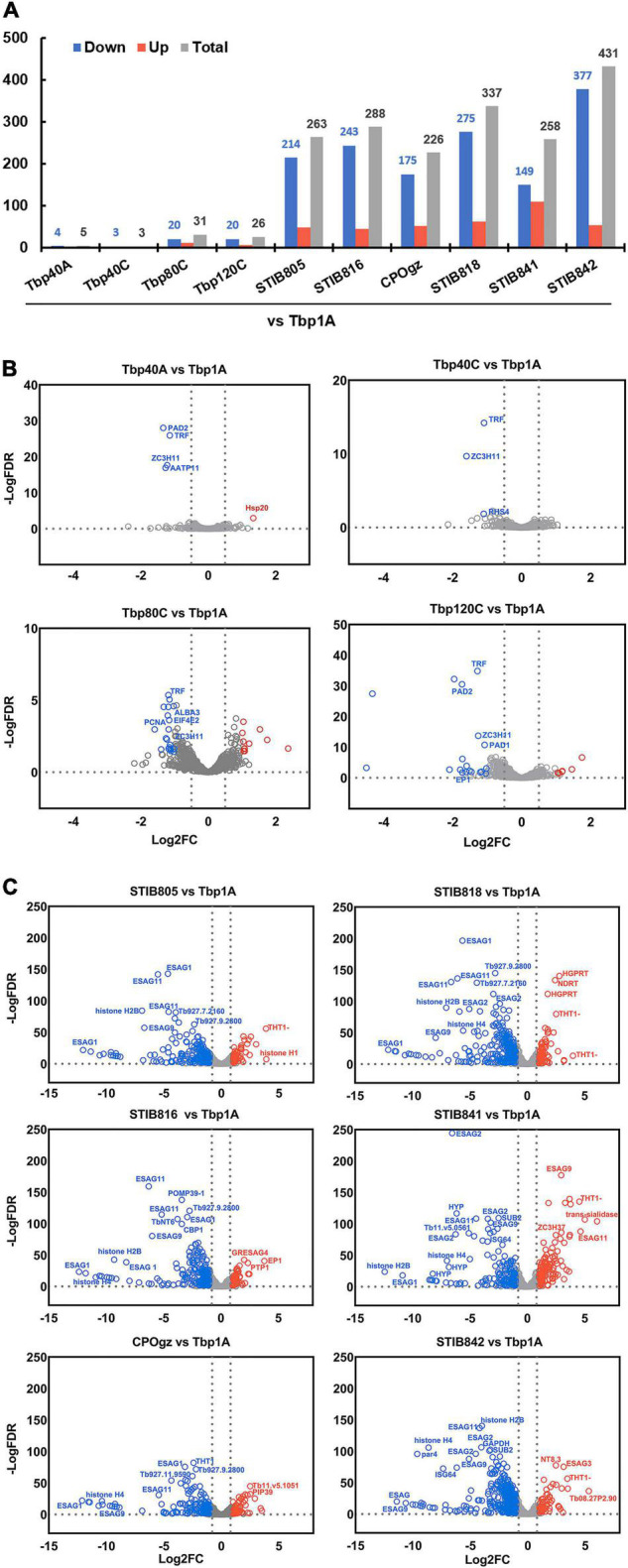
Differential expression of differentiation characteristic transcripts (DCTs) between pleomorphic *T. brucei* slender forms and monomorphic trypanosomes. **(A)** The histogram shows the number of statistically significant DCTs (≥2-fold, *q* ≤ 0.05) of all monomorphic trypanosomes relative to the pleomorphic *T. brucei* slender form (Tbp1A). Only a small proportion of DCTs significantly differed between the differentiation committed Tbp1A and laboratory-adapted monomorphic *T. brucei*; however, a considerable number of DCTs with significant differences in regulation were observed in the naturally occurred monomorphic *T. evansi* and *T. equiperdum* strains when compared with Tbp1A. **(B,C)** Volcano plot analysis of expression differences of DCTs between pleomorphic *T. brucei* slender form (Tbp1A) and monomorphic trypanosomes. Analyses highlighted the alterations in regulation that were significantly changed between Tbp1A and the laboratory monomorphic *T. brucei* **(B)**; naturally occurring monomorphic *T. evansi* and *T. equiperdum* **(C)**. The red labels show the Tbp1A down-regulated genes, while the blue labels indicate the opposite. Detailed results of these DCTs are showed in Supplementary Tables S4, S5.

Most of the DCTs remain at similar expression levels in the laboratory-adapted monomorphic trypanosomes as found in the differentiation competent BSF-LS (Tbp1A) (Figures 6A,B). Differences to Tbp1A were found in only 5, 3, 31, and 26 DCTs in Tbp40A, Tbp40C, Tbp80C and Tbp120C, respectively (Figure 6A and Supplementary Table S4), which is associated with the trend of the loss of differentiation. In terms of these identified changes, we only identified two DCTs that were consistently downregulated in the laboratory-adapted monomorphic trypanosomes, including the telomere DNA binding protein TRF1 and a zinc-finger domain-containing protein (ZC3H11) (Figure 6B). However, whether they are the direct cause of monomorphism still remains to be established.

Notably, many more changes have been observed in *T. evansi* and *T. equiperdum* strains, as 263, 288, 226, 337, 258, 431 DCTs were founded in STIB805, STIB816, CPOgz, STIB818, STIB841, and STIB842, respectively (Figure 6A and Supplementary Table S5). Among 263 genes in the comparison groups “Tbp1A vs. STIB805,” 84 BSF-SS-biased transcripts were significantly depleted in STIB805, including the genes coding for surface proteins such as. PADs, EP, GPEET, and EGAG9s etc. (Figure 6C), while corresponding groups compared with STIB816, CPOgz, STIB818, STIB841, and STIB842 exclusively contained, respectively, 42, 48, 79, 41, and 69 BSF-SS-biased transcripts. While a much smaller portion of BSF-SS-biased transcripts were uplifted (10, 34, 26, 34, 58, 35 in STIB805, STIB816, CPOgz, STIB818, STIB841, and STIB842, respectively). In term of transcripts favored by BSF-LS, 39, 11, 25, 28, 51 and 19 of them were particularly elevated in the naturally evolved monomorphic trypanosomes, such as the THT1-hexose transporter, probably reflecting a more vigorous metabolism; the ESAGs and histones were more biased toward *T. brucei* (Figure 6C), consistent with the surface proteins and cell cycle regulation during the differentiation process of *T. brucei*. However, the remaining 130, 201, 127, 196, 108, and 308 were, surprisingly, down-regulated in these naturally monomorphic trypanosomes, suggestive of a BSF-SS-like pattern.

**FIGURE 7:**
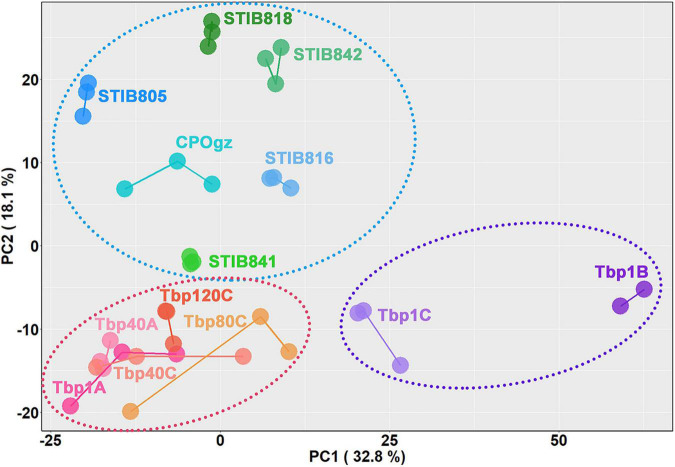
Relationships between differentiation potential of different trypanosomes and the expression of the selected differentiation characteristic transcripts (DCTs). Principal component analysis is shown for all analyzed DCTs. Samples are color-coded according to cell lines or differentiation stages, and individual samples are labeled on the plot. The dotted circles represent three different differentiation states: the purple one comprises samples containing stumpy forms, the red one comprises the monomorphic *T. brucei* established in the lab by rapidly passaging in mice as well as the slender forms in the pleomorphic trypanosomes, while the blue one comprises the naturally occurring monomorphic *T. evansi* and *T. equiperdum* strains.

Figure 7 shows the relationships between the differentiation potential of different trypanosomes and the expression of these differentiation characteristic transcripts (DCTs) based on principal component analysis (PCA) (Supplementary Table S3, Sheet 4). Three major trends were observed based on the two-dimensional representation of 50.9% DCTs variances: (i) The replicates of the biological samples collected from different infected mice were reliable, because they were most closely clustered. (ii) The differentiation process followed a consecutive progression from BSF-LS (upper left; Tbp1A) to BSF-SS (lower left; Tbp1B), as represented by PC1, while the intermediate proportion of BSF-LS and BSF-SS in Tbp1C (between Tbp1A and Tbp1B) was precisely reflected. (iii) The natural monomorphic *T. evansi* and *T. equiperdum* strains clearly settled into a distinguishable group in the upper left panel by PC2, while all the *T. brucei* AnTat1.1 cell lines were located at the bottom section.

### Expression Differences in Differentiation Regulatory Genes Between Differentiation-Committed *Trypanosoma brucei* Slender Forms and Monomorphic Trypanosomes

Interestingly, a considerable number of upstream differentiation regulators (between BSF-LS/SS and PCF stages) showed constant expression during the transition from BSF-LS to BSF-SS and fall out of the DCTs described above. Therefore, we explored the changes in the upstream regulatory network in monomorphic trypanosomes, including 40 genes related to the QS pathway and another 23 regulators (Supplementary Table S6, Sheet 1).

**FIGURE 8:**
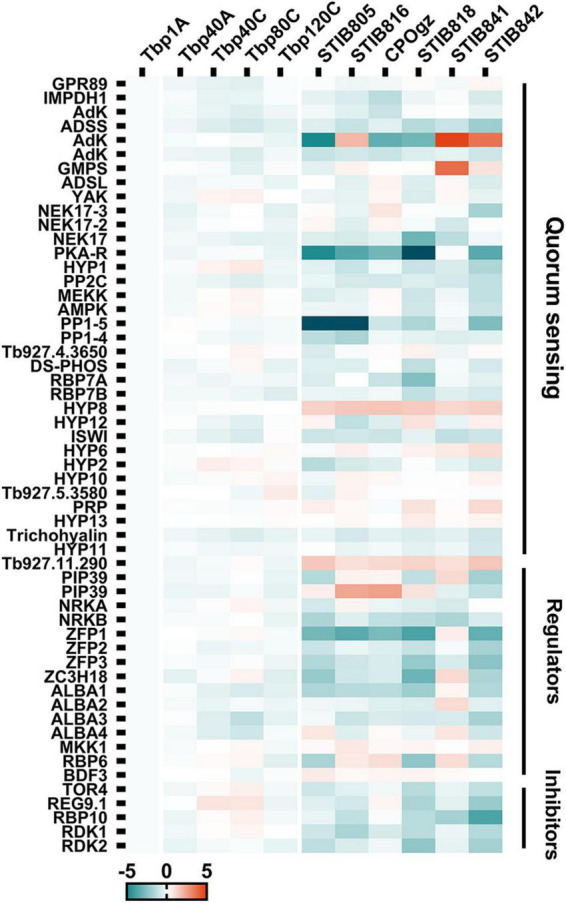
The heat map shows the relative expression of well-characterized differentiation regulatory genes (DRGs) in trypanosomes. Genes identified from the QS pathway, and the well characterized positive and negative regulators for generating stumpy forms, showed expression differences between pleomorphic *T. brucei* and monomorphic trypanosomes. Detailed changes in these DRGs are shown in Supplementary Figure S6.

Although the laboratory-adapted monomorphic *T. brucei* were incapable of differentiation into stumpy cells in mice, only a few possible changes were observed in these regulators that could explain the development of laboratory-adapted monomorphism. Regardless of the change in expression multiples, we focused on expression differences that were statistically significant (*q* ≤ 0.05) between the pleomorphic slender forms and monomorphic trypanosomes. We found that there were still some slight, but interesting differences, although these changes were not consistent in all passages (Figure 8; Supplementary Figure S5; and Supplementary Table S6, Sheet 2). In view of the statistical significance, 8 genes were associated with differentiation regulation in Tbp80, 15 genes in Tbp120, but none in Tbp40 stood out (Supplementary Figure S6). Compared with Tbp1A, two negative regulators (target of rapamycin kinases, TOR4), REG9.1 in Tbp80 were up-regulated with a 1.5-fold and 1.8-fold increase, respectively; but a gene (Tb927.11.6600) in the QS pathway was also controversially upregulated. The five down-regulated genes in Tbp80 including a SIF signal receptor GPR89, a transcriptional silencer imitation (ISWI), ALBA3/4 and a gene Tb927.11.11480. The 15 genes in Tbp120 included two unexpected up-regulated QS genes, Tb927.11.1640 with a 1.4-fold and Tb927.5.3580 with a 1.6-fold change; 13 genes with only a mild reduction (≤ 1.5-fold) including the eight genes involved in QS pathway (ADSL, ADSS, RBP7B, DsPhos, NEK17 (Tb927.10.5950), PP2C, PKA-R, and again Tb927.11.11480), and other five regulators (ZC3H18, ALBA1/3, RBP6, RDK2).

By contrast, the expression of genes involved in differentiation regulation appears to be extensively disturbed when comparing *T. evansi* and *T. equiperdum* (Figure 8; Supplementary Figure S5; and Supplementary Table S6, Sheet 3). Compared to Tbp1A, 16, 13, 12, 24, 11, and 27 transcripts were identified with significant changes (≥ 2-fold, *q* ≤ 0.05) in STIB805, STIB816, CPOgz, STIB818, STIB841 and STIB842, respectively. Additionally, there are 11, 15, 9, 10, 11, and 11 transcripts that have 1.5∼2-fold changes (*q* ≤ 0.05). What is interesting is that we found that most of the differentiation regulators were more highly expressed in *T. brucei* than in naturally evolved monomorphic *T. evansi* and *T. equiperdum*. Here, 22, 22, 16, 28, 11, and 33 regulators were down-regulated in STIB805, STIB816, CPOgz, STIB818, STIB841 and STIB842, respectively; while only a small number of genes were up-regulated, with 5, 6, 5, 6, 11, and 5 genes in each strain in turn (Supplementary Figure S6).

Significant down-regulation in differentiation regulator genes were observed in different *T. evansi* and *T. equiperdum* strains (Figure 8 and Supplementary Figure S6). Among them, 3 transcripts shared the same pattern of expression in all *T. evansi* and *T. equiperdum*, including two unexpected up-regulated hypothetical proteins (Tb927.11.300, Tb927.11.290) related to the QS pathway and one downregulated gene, ZFP3, a zinc finger protein required by pleomorphic *T. brucei* to promote BSF to PCF. In addition, we found 10 positive regulators that were down-regulated in most of the samples, of which 6 genes (PP1 (Tb927.4.3630), PKA-R, ISWI, NRKB, ALBA1/3) occur in 5 strains and 4 (AdK (Tb927.6.2360), ADSS, conserved protein (Tb927.11.6600), PP2C) are in 4 strains, but they were also accompanied by down-regulation of negative regulators RDK1, RDK2 and RBP10. Another 7 positive regulatory genes were found with a decreased expression trend in half of the samples, including 6 genes in the QS pathway: IMPDH1, NEK17 (Tb927.10.5950), MEKK, RBP7A, Tb927.9.4080, Tb927.11.11480, and zinc finger protein (ZFP2), while two negative regulator genes (TOR4, REG9.1) were down-regulated. 16 genes were changed in only one or two samples. The remaining 10 regulators were not consistent in the tested samples. These described changes may be sufficient to lead to a defective differentiation regulatory network, contributing to a fixed monomorphic state.

## Discussion

Cell differentiation is a typical characteristic of eukaryotic organisms from protozoans to mammals and, of course, including humans. This can be evidenced by the development in multicellular organisms which involves sequential differentiation events, from the zygote, through embryonic stem cells to fully formed individuals (Pourquié, 2018). It is also seen in protozoans with complicated life cycles, such as *Trypanosoma brucei*, the pathogen causing African human sleeping sickness or Nagana disease in livestock and wild mammals (Cayla et al., 2019). However, in multicellular organisms, malignancy can be triggered when disruption of differentiation happens to frequently dividing cells, such as epithelial cells, due to the accumulation of gene mutations (Aguilera and García-Muse, 2013). Theoretically, this phenomenon could occur in any eukaryotic including protozoans if they have a life cycle that promotes continuous proliferation. Previously, Lun and his colleagues have proposed that *Trypanosoma evansi* is a form of malignancy in *T. brucei*, while the RH strain is a malignant form of a wild type of the protozoan parasite *Toxoplasma gondii* (Lun et al., 2015). The key evidence supporting this notion is that these parasitic protozoans have lost their complicated cell differentiation processes and display sustainable proliferation similar to those found in typical malignant cells, such as leukemias, in metazoans. However, there is still a question as to how these malignant parasites are formed, whether there is any genetic relationship between these malignancies suggested in protozoans and those found in metazoans.

Through rapid passaging of pleomorphic *T. brucei* in mice, we demonstrated that pleomorphic trypanosomes gradually became monomorphic and produced a state that is similar to the phenotype found in *T. evansi* (Figure 1) and described previously (Turner, 1990). *T. evansi* is a naturally monomorphic trypanosome and has been suggested as a subspecies or even a mutant of *T. brucei* (Hoare, 1972; Lai et al., 2008). This trypanosome cannot differentiate into either the stumpy form in the bloodstream stage or the procyclic form in the insect vector (Supplementary Figure S3) and retains only the slender form which can sustain proliferation in the mammalian host. The monomorphic descendants of pleomorphic *T. brucei* we generated in the laboratory behave like *T. evansi*: they have uncontrolled growth in the host maintained by the absence of the G0-arrested point in the cell cycle. At the same time, the shortened survival time of infected hosts reflects their increased virulence when compared with survival times in the parental pleomorphic *T. brucei*. These phenomena mirror the process that occurs in cancer cells which transform from non-cancerous cells in metazoans (Lun et al., 2015). Additionally, the reduced efficiency of transformation from BSF into PCF, evidenced by the induced differentiation experiment *in vitro* (Figure 2), also indicates that the passaged monomorphic *T. brucei* have lost normal phenotypic differentiation. By comparing their transcriptomes, our results revealed that neither the monomorphic trypanosomes established in the laboratory (Tbp40, Tbp80 and Tbp120) nor the strains of *T. evansi* and *T. equiperdum* are similar to the differentiated *T. brucei* BSF-SS. However, the transcriptomes of the former still resemble differentiation committed *T. brucei* BSF-LS, while *T. evansi* and *T. equiperdum* are obviously transcriptionally distinct.

Although many studies, using generic genetic markers, show that genetic relationships between *T. evansi* and *T. equiperdum* and other *Trypanozoon* species remain highly conserved overall, mutations in the nuclear and mitochondrial genomes have been identified in *T. evansi* and *T. equiperdum* (Lun et al., 1992; Lai et al., 2008; Carnes et al., 2015). Here, our overall transcriptional analysis results demonstrate that they are distinct from *T. brucei* (Figure 4). Similar differences in post-transcriptional or post-translational regulation have also been found in other *T. evansi* strains (Zheng et al., 2019; Zhang et al., 2020). These changes could be the result of long-term evolution in nature. However, these established mutations have not been shown to be linked to *T. evansi* or *T. equiperdum* monomorphism (malignancy). These may be comparable to the abundant passenger mutations, described in human cancers, which have no direct or indirect effect on the selective growth advantage or malignant processes within the cell and are, probably, just related to the time that has elapsed between successive clonal expansions (Vogelstein et al., 2013), or to maintain the stability of monomorphism.

Recently, Schnaufer’s team has demonstrated that the key mutations in kDNA are not the primary cause for generating monomorphism in *T. evansi*. Instead, they proposed a SIF-independent background differentiation or inefficient SIF-dependent differentiation that may lead to the loss of stumpy differentiation (Dewar et al., 2018). Encouragingly, our detailed comparisons of the differentiation transcriptomes between the pleomorphic *T. brucei* and monomorphic *T. brucei*, *T. evansi* and *T. equiperdum* have identified key changes that could be the “driver” genes for regulating the cell fate of *T. evansi* (or *T. equiperdum*). We found that severe impairments of the well-explored differentiation regulatory pathway, which has been shown to be necessary for the full *T. brucei* life cycle, are observed in all monomorphic *T. evansi* and *T. equiperdum* strains. These may directly block the perception or transduction of the differentiation signal as well as the execution of the differentiation process in these pathogens, resulting in them being permanently locked in a monomorphic state, as opposed to the inducible laboratory-adapted monomorphic *T. brucei*. More interestingly, most of the crucial genes involved in differentiation regulation, in particular those genes involved in the quorum sensing (QS)-dependent cyclic adenosine monophosphate (cAMP) signaling pathway, are significantly down-regulated in *T. evansi* and *T. equiperdum*. The decrease shown in the expression of these genes, for example RBP7, DsPhos, NEK17, MEKK1 and PP1, are in good agreement with previous reports where the use of RNAi-mediated down-regulation or gene knockout on these regulators caused monomorphism in *T. brucei* (Mony et al., 2014; McDonald et al., 2018).

Vassella and colleagues had proposed that the inability of monomorphic trypanosomes to develop as BSF-SS was defective in the density sensing pathway (i.e., QS pathway) rather than defective in the differentiation process itself (Vassella et al., 1997). In our study, most of the transcriptome alterations found during the creation of our monomorphic mutants reflect the differentiation process differences between BSF-LS and BSF-SS, which are consistent with the physiological and morphological differences between the two stages. Thus, in terms of the above-mentioned alterations, it is not surprising that there were broad similarities in expression between the morphologically monomorphic and the pleomorphic slender forms. In fact, we observed only moderate growth inhibition by cAMP analogs in monomorphic *T. brucei* (Figure 3), as reported before by Hesse et al. (1995), Vassella et al. (1997), they found that the monomorphic parasites showed less sensitivity to conditioned medium (Hesse et al., 1995). This is also consistent with the reported results obtained using monomorphic trypanosomes produced *in vitro* (McWilliam et al., 2019). Thus, we expected to find SIF signal transduction defects in our study. However, the related genes for such defects were not detected in our transcriptome analysis. Such similarity is also supported by the global transcription analysis between monomorphic laboratory-adapted parasites and new isolates of *T. b. rhodesiense* from humans (Mulindwa et al., 2018), suggesting that changes at the molecular level do not occur easily, and may lag behind phenotypic changes.

In our study, we have investigated some of the detail behind the development of monomorphism. It is clear that there is a gradual loss of developmental competence in the monomorphic *T. brucei* strains, as evidenced by some easily overlooked observations. Tbp40 and Tbp80, the passaged trypanosomes, lost their ability to generate stumpy forms in mice, but this didn’t occur in rats, although the effect was weaker than seen in mice (Supplementary Figure S2). This observation is consistent with previous reports, in which 30∼50 rapidly syringe-passaged trypanosomes produced only slender forms in mice but they could still produce stumpy forms in cattle (Black et al., 1983). Additionally, there was only a slight accumulation of transcriptional changes observed in these monomorphic *T. brucei* as the passage number increased. One explanation for this could be that the population of these trypanosomes consisted of a proportion of cells that had a delayed transformation to BSF-SS, which was not observable before the host died due to overgrowth of the BSF-LS (Tasker et al., 2000). Another possibility might be that as the frequency of mechanical transmission increased, stumpy-committed slender populations were gradually replaced by rapidly proliferating cells, but the uncommitted slender populations remained to maintain a balance of infectious and transmissible parasite populations (MacGregor et al., 2012). A related explanation could also be that transformed cells (i.e., stumpy forms) exist below the detection threshold and this might explain why the occasional appearance of stumpy trypanosomes can be observed in different host individuals. In the literature, some support for this can be found in the observations that intermediate and short stumpy forms corresponding to those of *T. brucei* have been uncommonly reported in the monomorphic *T. evansi* in heterologous mammalian hosts (Hoare, 1956; Lun and Vickerman, 1991; Misra et al., 2016). Taken together, we put these speculations down to the advantages of cell growth, and speculate that the initiation of malignant *T. brucei* under laboratory simulations may not a one-step event.

In general, the monomorphic trypanosomes established in our study clearly represent a series of snapshots from pleomorphism to monomorphism. However, only two substantive phenotypic changes were observed, one is the loss of the ability to differentiate into stumpy forms, the other is the loss of proliferation control. Both of these phenomena are also observed in the naturally monomorphic trypanosomes, *T. evansi* and *T. equiperdum*. Putting together the data from the laboratory created monomorphic *T. brucei* and *T. evansi*, *T. equiperdum*, we think that they may represent quantitative and qualitative changes in the evolution of malignancy from pleomorphic *T. brucei*, respectively. It is well known that the more frequently the cell replicates, the greater the chance of genetic mutation (Macheret and Halazonetis, 2015). Thus, the development of malignant trypanosome cells could be based on quantitative increases in mutational changes which lead to qualitative changes in the phenotype of pleomorphic *T. brucei* BSF-LS to generate monomorphic forms. Theoretically, this process could be simulated in the laboratory using mechanical passage as we have initiated in this study. The outcome could be a gradual malignant process starting with epigenetic changes in expression that lead to more permanent transcriptional changes and eventually genetic changes that become fixed in the trypanosome cell population. This evolutionary process in trypanosomes is analogous to a tumor evolving from benign to malignant cells by acquiring a series of mutations over time, a process that has been particularly well studied in colorectal tumors (Nowell, 1976; Fearon and Vogelstein, 1990; Vogelstein et al., 2013); and is also in line with the reasons why pediatric cancers have fewer mutations than adult tumors (Milholland et al., 2015). Alternatively, the first qualitative stage could be the occurrence of key mutations that affect differentiation and promote monomorphism and malignancy, followed by the longer-term accumulation of genetic changes (quantitative stage) that differentiate species such as *T. evansi* and *T. equiperdum* from *T. brucei*. Thus, our results also provide strong support for the initial evolution of malignancy in trypanosomes. Firstly, there is selection for growth dominant forms leading to the emergence of monomorphic trypanosomes. This is a more extensive phenomenon that can be relatively easy to achieve through short-term simulations (as in the emergence of laboratory-adapted monomorphic *T. brucei*). This then sustains growth of malignant monomorphic trypanosomes favoring the development of alternative transmission routes (as occurs in *T. evansi* or *T. equiperdum*) by increasing the overall parasitemia (Desquesnes et al., 2009). Secondly, loss of cyclical development further reduces the necessity to retain genes essential for developmental regulation (e.g., the transcriptional changes found in this study) or survival at the vector stage (e.g., loss of mitochondrial genome in *T. evansi* or *T. equiperdum*).

From a disease epidemiological and evolutionary perspective, this may raise some concerns. In 2020, the World Health Organization achieved their target of elimination of human sleeping sickness from Africa (Gao et al., 2020). However, some concerns have been raised about the sustainability of elimination (Mehlitz and Molyneux, 2019) and there are key concerns about possible resurgences due to unexpected epidemiological factors. Our study suggests another possible concern: the *de novo* generation of human infective trypanosome strains and novel epidemiological cycles. This could occur by human infective strains, such as *T. b. rhodesiense* or *T. b. gambiense* evolving monomorphism and the development of mechanical or venereal transmission routes between humans. Alternatively, pre-evolved monomorphic trypanosomes (e.g., *T. evansi*) could evolve human infectivity - as has already been reported (Truc et al., 2013). It is important, therefore, to fully understand the process of development of monomorphism in *T. brucei*.

The evolution of malignant trypanosomes clearly happens, and obviously, it can be quite common, occurring both in the laboratory and in the field. Evidence has been obtained, which demonstrates that the differentiation process, from the proliferating BSF-LS to BSF-SS in *T. brucei*, occurs through QS in response to the hormone-like stumpy induced factor (SIF), in which the signal is mainly transduced via a cAMP signaling pathway (Vassella et al., 1997). In fact, this conserved signaling pathway plays pivotal roles in cell signaling across eukaryotes and regulates many physiological and pathological processes (Sassone-Corsi, 2012). It has both tumor-suppressive and tumor-promoting roles (Cho-Chung et al., 1995; Fajardo et al., 2014). In addition, in most eukaryotic cells, the target of rapamycin (TOR) and AMP-activated kinase (AMPK) pathways are representatives of diverse, versatile energy sensors and metabolic regulators that regulate cell fate decisions (Inoki et al., 2003; Xu et al., 2012; Hart et al., 2015; Carling, 2017). Similarly, *T. brucei* has also been shown to manipulate these pathways in order to achieve a fine-tuned balance between energy consumption and the differentiation processes (Barquilla et al., 2012; Saldivia et al., 2016). It turns out that these biochemical or developmental pathways are highly related both in protozoa and metazoans, and as such, may provide a potential starting point for analogous studies in human cells. The vast majority of our knowledge of cancer driver genes has been derived from the study of the pathways through which their homologs work in non-human organisms (Vogelstein et al., 2013). Here, the single-celled trypanosomes, with their superior differentiation plasticity, may also provide an excellent model for exploring cell fate decisions and may help revealing the origins, evolution and occurrence of metazoan malignancy.

## Data Availability Statement

The datasets presented in this study can be found in online repositories. The names of the repository/repositories and accession number(s) can be found below: NCBI GEO, accession no: GSE184265.

## Ethics Statement

The animal study was reviewed and approved by the Sun Yat-sen University under the license no 31720103918.

## Author Contributions

Z-RL and D-HL contributed to conception and design of the study, guided the experimental operation, and analyzed the data. X-LC, S-JL, and PZ performed the experiments. X-LC performed the statistical analysis and wrote the first draft of the manuscript. All authors contributed to manuscript revision, and approved the submitted version.

## Conflict of Interest

The authors declare that the research was conducted in the absence of any commercial or financial relationships that could be construed as a potential conflict of interest.

## Publisher’s Note

All claims expressed in this article are solely those of the authors and do not necessarily represent those of their affiliated organizations, or those of the publisher, the editors and the reviewers. Any product that may be evaluated in this article, or claim that may be made by its manufacturer, is not guaranteed or endorsed by the publisher.

## Footnotes

1
https://tritrypdb.org/common/downloads/Current_Release/TbruceiTREU927/gff/data/

2
www.graphpad.com
