# Supplementary material for: The Occurrence of Malignancy in Trypanosoma brucei brucei by Rapid Passage in Mice
Source: Front Microbiol. 2022 Jan 11;12:806626. doi: 10.3389/fmicb.2021.806626 (PMC8789148; doi:10.3389/fmicb.2021.806626)
Supplement: Supplementary file 1 [file Data_Sheet_1.docx]

Supplementary Materials

**

**Supplementary Figure S1.** Strains of *Trypanosoma* spp. investigated in this study. NA, information not available.

**
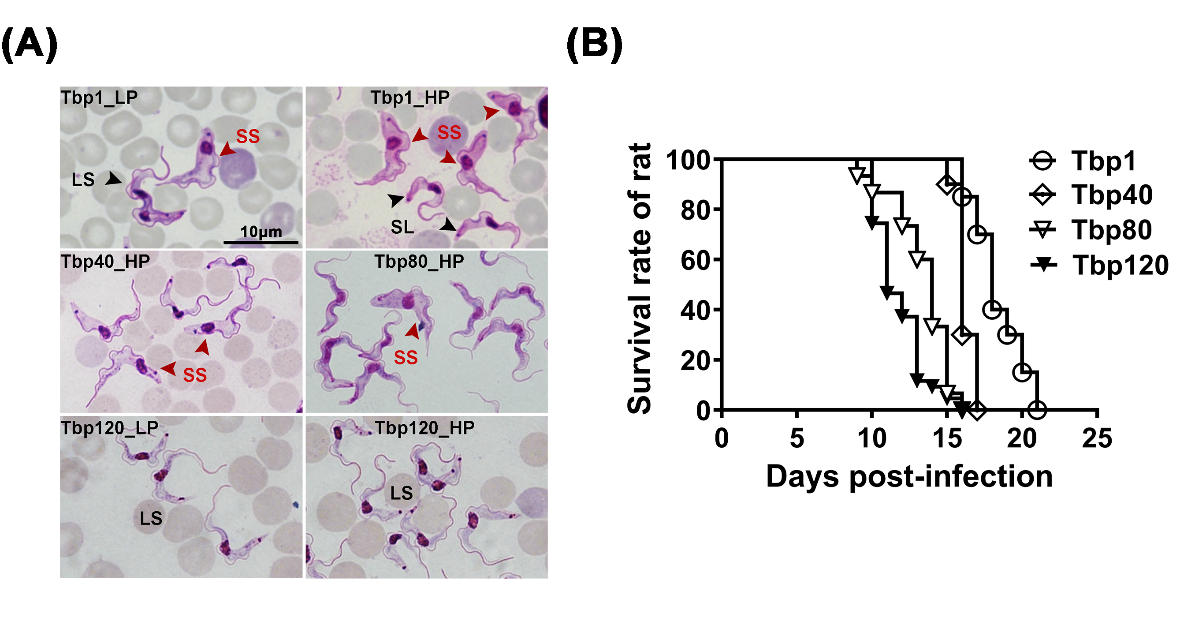
**

**Supplementary Figure S2.** The differentiation potential and virulence of different passaged populations of *T. brucei* in rat infections. **(A)** Giemsa-stained morphology of passaged *T. brucei* in rat infection. **(B)** The survival of the rats infected with different passaged *T. brucei*.

**
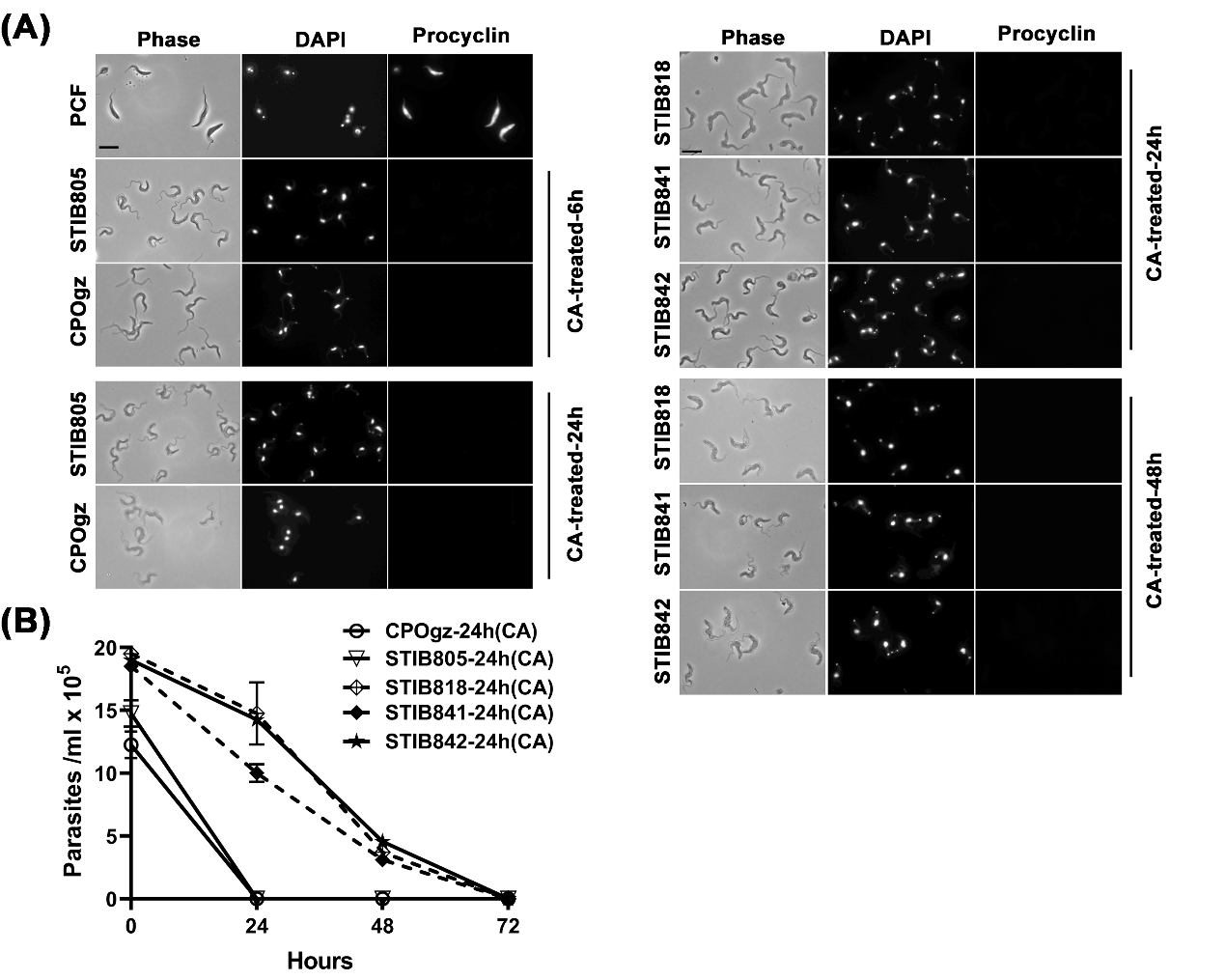
Supplementary Figure S3.** Transformation ability of different strains of *T. evansi and T. equiperdum* from bloodstream stages into procyclic stages *in vitro*. **(A)** Expression of the procyclic stage-specific coat procyclin of *T. evansi and T. equiperdum* strains treated with cis-aconitate (CA) for different times *in vitro*. **(B)** The growth status of trypanosomes after withdrawal of CA from the medium for different hours.


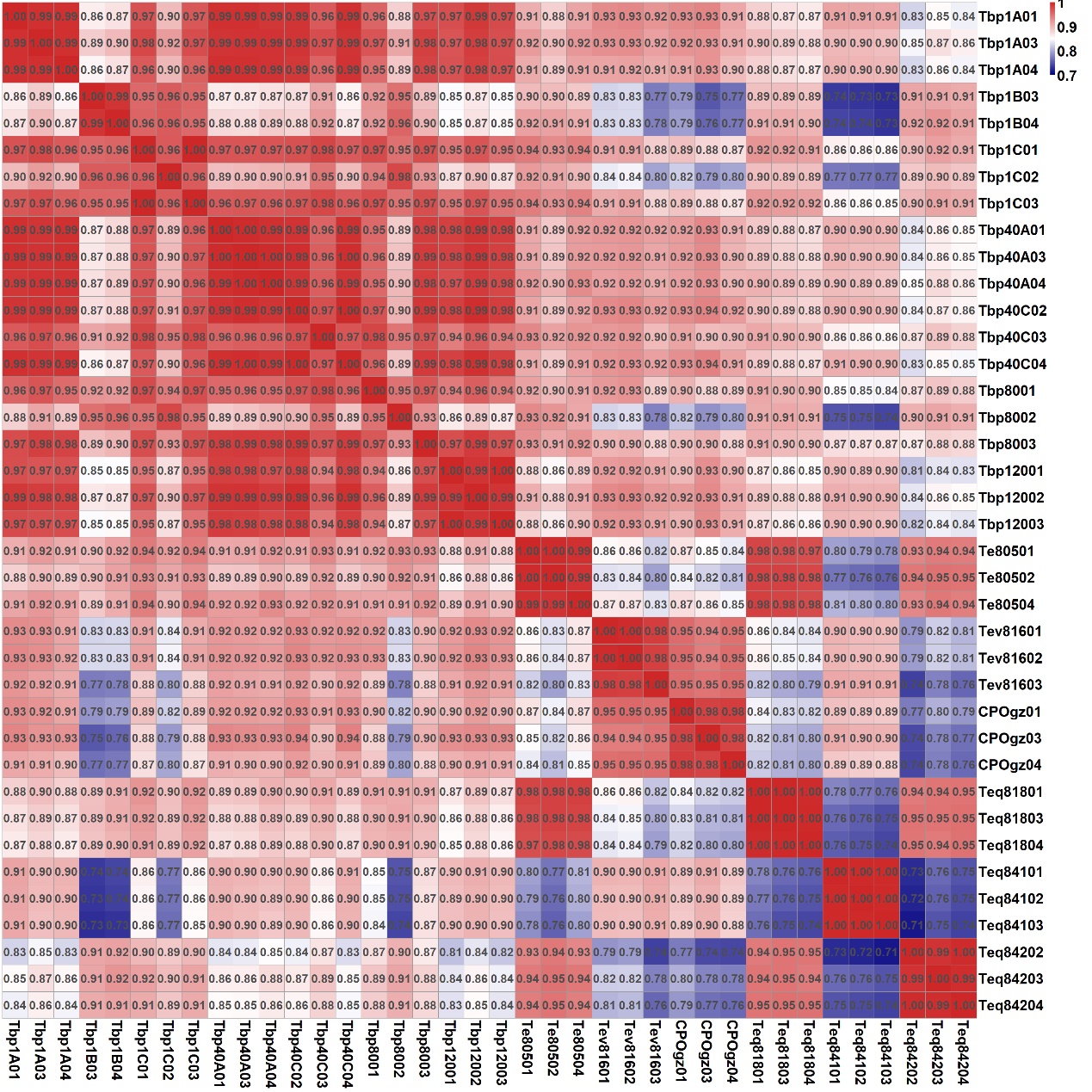
**Supplementary Figure S4.** Correlation of biological replicate samples. Pearson correlation values are shown for comparison groups.

The number of DEGs compared between the *T. brucei* slender and stumpy forms.


The number of DEGs between pleomorphic *T. brucei* (Tbp1A, Tbp1B) and monomorphic cell lines.

**Supplementary Figure S5.** The number of differentially expressed genes (DEGs; FC ≥ 2-fold, q ≤ 0.05) in all comparison groups.

**Supplementary Figure S6.** The expression differences of differentiation regulatory genes (DRGs) of all monomorphic trypanosomes relative to pleomorphic *T. brucei*. Note: Functional stage: QS and positive regulators are marked in black; negative regulators are marked in red. Fold change: The predicted significant changes that will inhibit differentiation are marked in red; if not, marked in black.

**Supplementary Table S1.** Primers for qPCR analysis investigated in this study. (XSLX)

**Supplementary Table S2.** Genome alignment and gene expression information for the samples. Summary of the mapping rate of transcriptome to genome (sheet 1) and expression values (Fragments Per Kilobase per Million, FPKM; sheet 2) for all transcripts in all samples. (XSLX).

**Supplementary Table S3.** Differential expression of transcripts between *T. brucei* slender and stumpy forms. Functional assignment for the significantly regulated transcripts (≥ 2-fold, q ≤ 0.05) among three samples (Tbp1A, 1B, 1C) of pleomorphic *T. brucei* (sheet 1). A total of 1026 differentially expressed genes between the *T. brucei* slender and stumpy were summarized in this study (sheet 2). The merged 1096 differentiation characteristic transcripts (DCTs) (sheet 4) that were statistically significant (q ≤ 0.05) and at least 2-fold different between *T. brucei* slender and stumpy forms in our study (sheet 2) or Silvester et al. (2018) (sheet 3). (XSLX)

**Supplementary Table S4.** Differential expression of differentiation characteristic transcripts (DCTs) between pleomorphic *T. brucei* slender forms and laboratory-adapted monomorphic *T. brucei*. Transcripts with significantly regulation differences between *T. brucei* slender and stumpy forms (e.g. DCTs) that showed statistically significant (≥ 2-fold, q ≤ 0.05) expression differences between the differentiation competent *T. brucei* slender form and laboratory-adapted monomorphic *T. brucei*. (XSLX)

**Supplementary Table S5.** Differential expression of differentiation characteristic transcripts (DCTs) between pleomorphic *T. brucei* slender forms and *T. evansi*, *T. equiperdum*. Transcripts with significantly regulation differences between *T. brucei* slender and stumpy forms (e.g. DCTs) that showed expression differences (≥ 2-fold, q ≤ 0.05) between the differentiation competent *T. brucei* slender form and monomorphic *T. evansi*, *T. equiperdum*. (XSLX)

**Supplementary Table S6.** Transcriptional differences of differentiation regulation genes (DRGs) between pleomorphic slender and monomorphic trypanosomes*.* 63 genes identified from the QS pathway and a summary of the well characterized positive and negative regulators for generating stumpy forms (sheet 1). Transcripts with significant transcription differences (q ≤ 0.05) between pleomorphic slender and laboratory-adapted monomorphic. *T. brucei* (sheet 2). Transcripts with significant transcription differences (≥ 2, q ≤ 0.05) between pleomorphic slender and *T. evansi* and *T. equiperdum* strains (sheet 3). (XSLX)
